# Detection and Treatment of *Helicobacter pylori*: Problems and Advances

**DOI:** 10.1155/2022/4710964

**Published:** 2022-10-22

**Authors:** Hang Yang, Liwen Guan, Bing Hu

**Affiliations:** ^1^Department of Gastroenterology, West China Hospital, Sichuan University, Chengdu, Sichuan, China; ^2^Department of Gastroenterology, Sanya Central Hospital (Hainan Third People's Hospital), Sanya, China

## Abstract

*Helicobacter pylori* (*H. pylori*) infection is chronic and etiologically linked to gastric cancer (GC) derived from gastric epithelium. The potential mechanism is complex, covering chronic inflammation, epithelial senescence, NF-*κ*B activation, the cytotoxin-associated gene A protein translocation, and related abnormal signaling pathways. In clinical practice, the test-and-treat strategy, endoscopy-based strategy, and (family-based) screen-and-treat strategy are recommended to detect *H. pylori* and prevent GC. It has been demonstrated that the decreasing annual incidence of GC is largely attributable to the management of *H. pylori*. This study reviews the current clinical practice of *H. pylori* on the detection and eradication, alternative treatment strategies, and related problems and advances, and hopes to contribute to the better clinical management of *H. pylori*.

## 1. Introduction

*Helicobacter pylori* (*H. pylori*) causes persistent infection in about 50% of the global population [1]. Gastric cancer (GC) occurs in 1–3% of *H. pylori* infected patients, accounting for 90% of all non-cardia GC (NCGC) and 20% of all cardia (CGC) cases, and *H. pylori* accounts for 15% of the global cancer burden [2–4]. Its infection may be associated with a sixfold increase in NCGC risk and a threefold increase in CGC risk [5]. In addition, all the precancerous conditions of GC including chronic atrophic gastritis (CAG), metaplasia, foveolar hyperplasia, and gastric hyperplastic polyps derived from the gastric epithelium are commonly caused by *H. pylori* infection [6]. Immunologically, it can evade host immune clearance and persistently colonize the niches, ultimately leading to the activation of pattern recognition receptors on antigen-presenting cells, gastric epithelial cells, and neutrophils [7]. In addition, *H. pylori* induces the activation of NF-*κ*B of gastric epithelial cells and leukocytes [7], contributing to the long-term colonization of *H. pylori*, chronic inflammatory microenvironment, and abnormal apoptosis, which further leads to accumulating mutations and malignant transformation of gastric epithelial cells [8, 9]. However, senescence associated with aging and chronic inflammation may contribute to the neoplastic transformation [9, 10]. Oncoprotein cytotoxin-associated gene A (*CagA*) is also a dominant factor leading to GC and activates oncogenic signaling pathways and inactivates tumor suppressors [11]. *H. pylori* detection and eradication strategies are effective for GC prevention, before considering GC endoscopic or barium photofluorographic screening, especially in areas with high GC risks [12, 13]. In addition, *H. pylori* may exert systemic pathological effects, although its infection is localized [13]. Strong associations have been established between *H. pylori* and some extra-gastric diseases, such as unexplained iron-deficiency anemia, idiopathic thrombocytopenic purpura, and vitamin B12 deficiency. It is also recommended to detect and eradicate *H. pylori* in patients with these diseases [14]. Additionally, *H. pylori* infection may be involved in neurological diseases [15], cardiovascular diseases [16], hepatobiliary diseases [17, 18], and autoimmune diseases [19]. More research is required to explore the possible associations and mechanisms, which may further contribute to the treatment and prevention of these diseases by *H. pylori* eradication.

## 2. *H. Pylori* Detection in Clinical Practice and Advances

The risk factors for *H. pylori* infection include age, ethnicity, levels of hygiene, and economic and social conditions [20, 21]. GC and its precancerous lesions are predominantly caused by *H. pylori* infection [6]. Timely diagnosis and subsequent eradication of *H. pylori* are recommended to abolish inflammation and may contribute to the regression of CAG and metaplasia, especially in mild or moderate severity [22]. Diagnoses are currently based on endoscopy and laboratory tests including urea breathing test (UBT; C^13^ UBT sensitivity 90–100%/specificity 94–100%), stool antigen test (SAT; sensitivity 73.9–95.0%/specificity 86.8–100.0%), serological test (sensitivity 76–84%/specificity 79–90%), endoscopy and biopsy-based diagnostic methods (histology (sensitivity 83–95.5%/specificity 95.4–100.0%), rapid urease test (RUT; sensitivity 85.0–99.0%/specificity 92.4–100%), and culture (sensitivity 67.9–96.0%/specificity 79.4–100.0%) [23, 24]. Method option is based on the prevalence rate of GC and *H. pylori* infection, patient wishes, endoscopic findings, and medical policies. For example, test-and-treat strategy is recommendable for uninvestigated dyspepsia including functional dyspepsia with non-invasive tests [25–28]. UBT remains an important tool in the test-and-treat strategy and post-eradication retest. SAT is considered as an alternative in detecting *H. pylori* and retest after eradication, as well as for children and patients after gastric surgery [29, 30]. Serological test can also be used for children and patients with peptic ulcer bleeding, gastric MALT lymphoma, severe gastric atrophy and intestinal metaplasia (IM), or the use of proton pump inhibitors (PPI)/antibiotics. The *H. pylori* endoscopy-based strategy with biopsies including RUT and histology is recommended in dyspeptic patients, older patients, or patients with alarm symptoms, especially in populations with low prevalence of *H. pylori* infection (<10%). Kyoto classification based on conventional white light imaging (WLI) is reported with more than 80% diagnostic accuracy rate [31]. Image-enhanced endoscopy (IEE) can enhance the visualization of gastric microstructures and also accurately diagnose *H. pylori* infection [32]. Furthermore, artificial intelligence (AI) models based on different endoscopic images including WLI and IEE images have been conducted and achieved a pooled diagnostic accuracy of 80% [33]. Regarding detecting antibiotic resistance, biopsy-based culture is recommended for antibiotic susceptibility test. Other advanced molecular methods include quantitative real-time polymerase chain reaction (qPCR) and digital PCR (dPCR). qPCR testing gastric biopsy samples, gastric juice, or stool has showed >90% sensitivity in *H. pylori* taction and 100% sensitivity when testing antibiotic resistance in patients with dyspepsia, which have similar or higher diagnostic ability than that of histological methods [34–36]. dPCR is found to detect “occult” *H. pylori* infection in a considerable proportion of patients with false negative results of conventional methods [37]. Maastricht VI/Florence Consensus Report recommends clarithromycin susceptibility testing, if available through molecular techniques or culture, before prescribing any clarithromycin containing therapy [14]. PCR or next-generation sequencing has powerful diagnostic ability and is recommended to test resistance [38–40]. The *H. pylori* screen-and-treat strategy is addressed in communities with high GC risks, especially in young adults without precancerous lesion, which is the most cost-effective [41]. For areas with high infection rate and related disease burden, this strategy is beneficial and cost-effective. As *H. pylori* will pass by oral cavity before entering the stomach and potentially transmit mostly among family members, especially during childhood and adolescence, family-based screen and treat strategy is recommended in the family with GC family history or gastric mucosal precancerous lesions [24]. *H. pylori* eradication can reduce GC risk in individuals who have a GC family history in first-degree relatives [42] and is cost-effective for GC prevention compared with no screening strategy [43]. Serological test, SAT, and UBT have been used in screening [44]. Rapid urine test may have similar screening ability with serological test. [45] Additionally, gastric functional serology (pepsinogens I/II and gastrin) may provide clinically information on CAG as a complementary diagnostic tool, although it is not recommended for *H. pylori* detection and screening [14]. For the efficiency and cost effectiveness, biosensors may be an alternative to directly and real-timely recognize elements of *H. pylori* including antigens (CagA, VacA, flagellin, and adhesins)/antibodies, oligonucleotides (DNA), and enzymes (urease enzyme) maybe via electrochemical/optical/piezoelectric/microfluidic based biosensor platforms, although challenges in the fabrication of biosensors exist, such as transducer selection, bio-recognition element, and their proper immobilization [46]. Volatile organic compounds from breath are also demonstrated to determine *H. pylori* infection from the view of metabolism [47, 48].

## 3. *H. Pylori* Eradication in Clinical Practice

Presently, chronic *H. pylori* infection can be cleared only by medical eradication treatment [49]. The annual incidence of GC and CAG has been decreased [50, 51], mainly due to the accurate detection and successful eradication of *H. pylori* [42, 52, 53]. The most common first-line regimens are the standard clarithromycin-based triple therapy (STT), bismuth-based quadruple therapy (BQT), and non-bismuth-based quadruple therapies (non-BQT; concomitant [CT], sequential, hybrid, and tailored therapy) [54]. STT is associated with antibiotic resistance and related to the low eradication rate [54]. The choice of STT should be guided by the local clarithromycin resistance rate and the use history of macrolides [55]. STT should be avoided when its resistance is >15%, or unknown clarithromycin resistance or local eradication rate is <80–85% [14, 56]. In addition, clarithromycin susceptibility can be tested by culture or PCR, when STT is considered as the first-line therapy, except in populations or regions with low clarithromycin resistance (<15%) [38, 57]. In areas with high clarithromycin resistance (>15%) or unknown clarithromycin resistance, BQT is recommended as the first-line therapy. In areas with high dual clarithromycin and metronidazole resistance, BQT is first recommended [24, 55, 58]. If BQT is not available, non-BQT may be considered. Patient nonadherence, duration of therapies, dosage of PPIs (the level of gastric juice pH), body mass index, optimal dosage of antibiotics, and number of drugs are associated with the efficacy of eradication treatment. For example, overweight and low level of gastric juice pH are found to reduce the eradication rate [59, 60]. Prolonged duration may be able to increase the eradication rate [61]. Related studies have focused on these issues to find alternative antibiotics regimens with better medical adherence. The choice of PPIs is essential to achieve higher intraluminal pH. The novel potassium-competitive acid blocker vonoprazan (VPZ) is shown to be more effective than lansoprazole in STT (93% vs 76%), even in patients with clarithromycin resistance (82% vs 40%) [62]. Another potential alternative therapy is high-dose amoxicillin–PPI dual therapy (3–4 times daily for 14 days), showing a higher or similar eradication rate ranging from 84.7 to 95.3%, compared with other first-line regimens [63–65]. Furthermore, VPZ-based dual therapy (VPZ 20 mg twice daily (bid) and amoxicillin 500 mg 2/3 times daily (bid/tid)) for 1 week achieve noninferior eradication rate to VPZ-based triple therapy (VPZ 20 mg bid, amoxicillin 750 mg bid and clarithromycin 200 mg bid for 1 week; 84.5% vs 89.2%/92.9% vs 91.9%), as well as similar adverse events (AEs) [66, 67]. The eradication rate in strains resistant to clarithromycin for VA-dual (VPZ 20 mg and amoxicillin 750 mg bid) is higher than that of VAC-triple (VPZ 20 mg, amoxicillin 750 mg, and clarithromycin 200 mg bid; 92.3% vs 76.2%) [66]. Apart from the alternatives of PPIs, rifabutin containing therapies have also been studied for the alternative role in STT or rescue therapy [68, 69]. Additionally, one study changes the way of antibiotics use from oral administration to endoscopic administration by washing pipe. Amoxicillin 300 mg, metronidazole 200 mg, and clarithromycin 100 mg are mixed with sucralfate suspension 60 mL (600 mg) and distilled water 120 ml and applied to gastric and duodenal mucosa. One single-dose medicament can immediately eradicate *H. pylori* through one endoscopic examination [70]. These regimens are comparatively convenient to implement or effective, and more clinical trials are required.

Antibiotic resistance has been increased in most WHO regions. >15% primary and secondary resistance rates to clarithromycin, metronidazole, and levofloxacin are reported in all WHO regions. Antibiotic resistance is a major driver of eradication failure. For example, resistance to clarithromycin is associated with the failure of clarithromycin-containing regimens (odds ratio: 6.97) [71]. One research has evaluated 21,533 patients receiving STT based on European Registry on *H. pylori* management (Hp-EuReg) and finds 23% pretreatment resistance rate to clarithromycin, 32% to metronidazole, and 13% to both. The modified intention-to-treat eradication rate is 81.5%. ≥90% eradication rate is only achieved by BQT or CT [72]. In Asia–Pacific region, 17% primary resistance rate for clarithromycin and 44% for metronidazole are reported. In addition, <80% eradication rate with clarithromycin-containing regimens is reported in countries with >20% clarithromycin resistance rate [73]. The mechanisms are related to mutational changes encoded chromosomally and disrupt the cellular activity of antibiotics through target-mediated mechanisms [74]. Some factors other than mutations can also affect eradication, such as drug uptake and/or efflux and biofilm and coccoid formation. Drug uptake and/or efflux has been reported in amoxicillin, levofloxacin, nitroimidazoles, and tetracyclines [74]. Biofilm-forming *H. pylori* is shown tolerant to multiple antibiotics including amoxicillin, clarithromycin, and tetracycline [75]. Coccoid forms with high fatty acid and cholesterol contents are reported with resistance to antibiotics [76]. These resistance mechanisms can be correlated with abnormal consumption of antibiotics, which are also used to treat other common infections [77]. Higher eradication failure rate of clarithromycin-containing regimen is found in patients with a history of previous macrolide longer-duration use [78] or those with the highest macrolide prescriptions [79]. One study finds >95% primary resistance might originate from the transmission of resistant bacteria, and resistance may decrease if macrolides are out of use for purposes other than *H. pylori* eradication [80]. Another study finds that the previously treated adult group have higher resistance rates than treatment-naive adult group for metronidazole (99.2% vs 78.4%), clarithromycin (58.3% vs 19.0%), and levofloxacin (52.3% vs 23.3%) [81]. With the development and popularization of PCR, like COVID-19 virus detection technique, drug susceptibility test may be routinely performed. Susceptibility guided therapy may be generally more cost-effective than empirical options, under the background of antimicrobial stewardship to control or reduce antibiotic resistance [82].

## 4. Problems after Eradication Treatment

When receiving eradication treatment, the most common problems are AEs induced by drugs. One meta-analysis based on Hp-EuReg has reported that 7% taste disturbance, 7% diarrhea, 6% nausea, and 3% abdominal pain are the most frequent AEs. The majority of AEs are mild (57%), 6% are severe, and only 0.08% are serious. The average duration is 7 days. The treatment compliance rate is 97%, and the discontinuation rate due to AEs is 1.3%. These AEs are usually mild with limited duration and without apparently interference on treatment compliance [83]. After receiving eradication therapy, some issues require more concerns including recurrence, the risk of non-variceal upper gastrointestinal bleeding (NVUGIB), and the risk of GC. Recurrence can occur due to recrudescence or reinfection [84]. The global annual recurrence, reinfection, and recrudescence rates are 4.3%, 3.1%, and 2.2%, respectively. Recurrence rate varies widely among continents (Europe 16% vs Africa 1%), countries (Turkey 21.3% vs Netherlands 0.2%), and economic development levels (developing countries 13% vs developed countries 2.7%) [84–86]. It may be associated with socioeconomic and sanitary conditions (food and water source, local prevalence of *H. pylori*, and living and eating habits), host factors (genetics and immune function, selection of therapeutic scheme, and missed medication doses), and organism factors (antimicrobial resistance, coccoid formation, and oral colonization) [84, 87–89]. Therefore, besides the best regimen option and health education, retest is a necessary step especially for patients with aforementioned risk factors. One study reports the retest rate is less than a quarter [90], maybe because of patients' medical compliance, ages, test convenience, or potential risk of indication diseases for *H. pylori* or follow-up system. Routine post-eradication retest and ensuring timely follow-up eradication are important and should be focused on.

It is recommended that patients with hemorrhagic peptic ulcer diseases should receive *H. pylori* eradication if *H. pylori* test is positive to promote the ulcer healing and prevent recurrence [91]. Delays in both treatment and retreatment may increase mucosa damage risk, such as erosion, peptic ulcer, and bleeding [92, 93]. However, NVUGIB is also a potential problem, particularly in elder patients receiving eradication treatment and other drugs co-prescription, such as antithrombotics and NSIADS. In addition, there is a positive correlation in frail elder patients between aging and polypharmacy/hyperpolypharmacy (≥5/≥10 drugs), which may contribute to frailty, thus creating a vicious circle [94]. The hospitalization risk for NVUGIB in patients taking aspirin and selective serotonin reuptake inhibitors is higher than that of non-users after *H. pylori* eradication [95, 96]. Patients in whom *H. pylori* has been eradicated also have a higher long-term risk of NVUGIB, particularly in elder patients (>45 years), which increased significantly after the first two years following eradication [97]. Hidden reasons, such as clarithromycin use, PPI discontinuation, gastric dysbiosis, recurrence, and dietary habits, may be involved [98]. Health education and appropriate gastric mucosa protectant are necessary [99].

Although *H. pylori* eradication reduces GC, the risk of GC after *H. pylori* eradication still exists. 0.21% of *H. pylori*-eradicated patients develops GC during a mean follow-up of 4.7 years [100]. There are several potential risk factors with unclear mechanisms. For example, medications including PPIs, aspirin, cyclooxygenase-2 inhibitors, statins, and metformin are reported to be involved in GC development after eradication treatment [101]. Existing atrophy/metaplasia at the time of eradication is related to GC carcinogenesis. Mild or moderate atrophy can be reversible after eradicating *H. pylori*. Atrophy can also progress to GC in some conditions, such as advanced chronic atrophy gastritis and accompanied incomplete metaplasia, invisible dysplasia, genetic alterations of stem cells, or epithelial–mesenchymal transition [22]. The reversibility of metaplasia is controversial. Spasmolytic polypeptide-expressing metaplasia is usually reversible, and IM seems to have a “point of no return” [6]. Additionally, chronic inflammation and other long-lasting changes in the gastric mucosa caused by *H. pylori* infection, such as mitochondrial changes, senescence, (epi)genetic alterations, and dysbiosis of gastric microbiome, contribute to GC independent of *H. pylori* [9]. In clinical practice, surveillance every 3 years is recommended in patients with atrophy and/or IM affecting both antral and corpus mucosa or at OLGA/OLGIM III/IV stage [102, 103]. Health education is equally important, as lifestyle factors will cause mucosal damage and contribute to GC carcinogenesis [104]. Some advances may aid our clinical practice. For example, endoscopic images can be diagnosed in real time by AI models to achieve “visual pathology” [105]. Gas biopsy and liquid biopsy to early diagnose GC from cellular metabolism and DNA level are under investigation, which may contribute to the diagnosis of invisible dysplasia and invisible residual/recurrent GC. Apart from early diagnosis, one machine learning model based on patients' baseline information predicts the risk of post-eradication GC [100]. Another machine learning model combines endoscopic and histologic findings at initial endoscopy to predict personalized risk of GC [106]. GC prediction is earlier than early diagnosis to predict GC in store.

## 5. Potential Alternative Treatment Strategies

**Figure 1:**
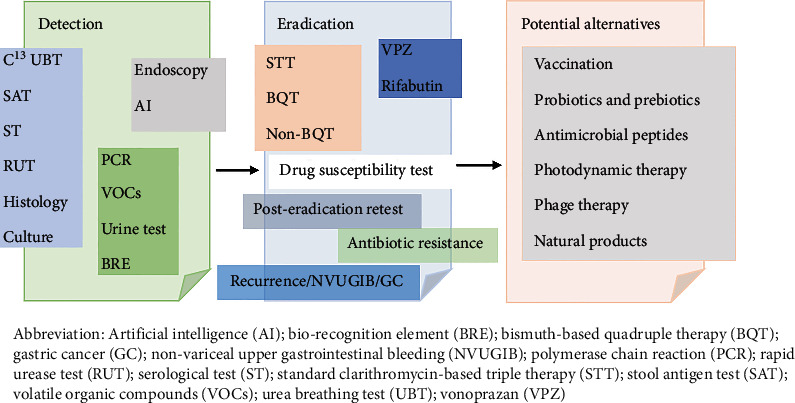
Detection and eradication of *Helicobacter pylori.*

*H. pylori* can efficiently evade innate immune detection and persistently colonize in the inhospitable environment with an acidic pH < 3 and a rapidly renewing gastric epithelium [107]. Successful eradication of *H. pylori* cannot fully ensure the continued protection [88]. Vaccination against *H. pylori* would be effective to achieve prophylaxis or eradication and reduce the prevalence of gastric diseases [108]. A prophylactic or therapeutic vaccine needs the selection and combination of immunogenic bacterial antigens with effective adjuvants and its administration via an available route [109]. The main antigens related to vaccines are CagA, VacA, BabA, HpaA, NapA, OipA, GGT, HspA, Omp, and FliD [110], and their combinations of multivalent epitopes with adjuvants, usually composed of CD4^+^ and CD8^+^ epitopes [111] (e.g., CTB-UE, CWAE vaccine [112], CFdAE vaccine [113], FVPE vaccine [114], HUepi-LTB vaccine [115], LHUC-LTB [116], and CTB-HUUC vaccine [117]). Some vaccines have been developed, such as oral delivery of either whole cell or subunit vaccines in combination with cholera toxin and *Escherichia coli* enterotoxin as mucosal adjuvant to increase the immunogenicity, oral delivery of live vector vaccines expressing *H. pylori* antigens to stimulate durable immunity (e.g., avirulent strains of *Salmonella* and *attenuated Listeria monocytogenes*), and intramuscular delivery of *H. pylori* subunits vaccines with aluminum hydroxide adjuvant [118–120]. *H. pylori* vaccines could reduce bacterial load and sometimes provide sterilizing immunity, whereas ineffective or only partially effective results were achieved in larger animals and patients [121]. Most vaccines are at a very early stage (phase I or even preclinical) lack of continuity and with inconsistent results [110]. However, a randomized phase 3 study with children indicated oral vaccines with recombinant urease B was efficacious and safe [122]. It is promising and inevitable in the future guideline. In addition to vaccines, a number of other potential alternative treatment strategies are being designed. Probiotics and prebiotics as adjuvants in *H. pylori* treatment are shown with higher successful eradication rate [123, 124]. Antimicrobial peptides, which contain 9 groups and 22 antimicrobial peptides, have anti-*H. pylori* effects including drug-resistant *H. pylori* by *α*-helical structure, being cationic, with high positive charge and isoelectric point [125]. They have the potential to replace the antibiotics and limit the spread of antimicrobial resistance of *H. pylori* [126]. Photodynamic therapy (PDT) oxidizes biomolecules and causes irreversible damage through reactive oxygen species production by a photosensitizer (PS) under laser irradiation. PDT can kill *H. pylori* with/without drug resistance. A *H. pylori*-targeted PS can avoid undesirable phototoxicity to normal cells. Multiple 3SL conjugated poly-L-lysine based photomedicine with a *H. pylori* targeted PDT strategy using an endoscopic laser system has been proposed [127]. Phage therapy is also promising in eradicating *H. pylori*, and the use is still underdeveloped, such as the preparation of specific lytic phage [128]. Some studies also show that natural products, including fruits, vegetables, spices, and medicinal plants, have inhibitory effects on *H. pylori*, suggesting their potential as alternative options for the management of *H. pylori* infection [129] (Figure 1).

## 6. Conclusions

The chronic infection of *H. pylori* is usually acquired in childhood. They can safely colonize around the gastric glands and further induce chronic inflammation, NF-*κ*B activation, and CagA translocation, which cause damage and the loss of gastric glands and even neoplastic transformation and carcinogenesis. Therefore, the detection and eradication of *H. pylori* are necessary. The regimen option is currently based on antibiotic resistance and experience. Some problems after eradication require our concerns including recurrence, the risk of NVUGIB, and the risk of GC. Routine susceptibility test and retest, regular and careful endoscopic surveillance, and health education contribute to controlling or solving these problems to achieve better *H. pylori* management. Additionally, potential alternative treatments may aid the current antibiotics treatment. The development of vaccine may be one solution to achieve a wide range of preventive effects and eradication treatment.
